# CTX-M and Plasmid-mediated AmpC-Producing *Enterobacteriaceae*, Singapore

**DOI:** 10.3201/eid1006.030726

**Published:** 2004-06

**Authors:** Tse Hsien Koh, Grace Chee Yeng Wang, Li-Hwei Sng, Zhao Yi, Tse Yuen Koh

**Affiliations:** * Singapore General Hospital, Singapore;; † Ngee Ann Polytechnic, Singapore

**Keywords:** extended-spectrum β-lactamase, Escherichia coli, Klebsiella pneumoniae

**To the Editor:** In gram-negative bacteria, β-lactamases are an important cause of antimicrobial resistance. In the 1990s, several new β-lactamases, including CTX-M type and plasmid-mediated AmpC β-lactamases, emerged.

CTX-M extended spectrum β-lactamases (ESBLs) differ from those derived from TEM and SHV enzymes by their preferential hydrolysis of cefotaxime and ceftriaxone compared with ceftazidime. They also differ from an evolutionary standpoint and are more closely related to the chromosomal enzymes of *Kluyvera* species (*1*). These enzymes are increasingly described worldwide, particularly in South America, Europe, and East Asia.

AmpC enzymes confer resistance to oxyimino- and 7-α-methoxy-cephalosporins. They occur naturally in the chromosomes of bacteria such as *Enterobacter*, *Citrobacter*, *Serratia*, and *Pseudomonas* species. In the last decade, genes coding for AmpC β-lactamases have made their way into plasmids and are increasingly detected in other species, notably *Klebsiella* and *Escherichia coli* (*2*).

We describe five strains of *Enterobacteriaceae* with unusual antimicrobial susceptibility patterns, which were isolated from patients in an 800-bed hospital in Singapore. *K. pneumoniae* EU17113, EU2673, and *E. coli* EU2657 were noted to be more susceptible to ceftazidime than ceftriaxone, whereas *E. coli* EU4855 and EB9505 were resistant to both cephalosporins. The National Committee for Clinical Laboratory Standards ESBL confirmatory test (*3*) was positive for strains EU17113, EU2673, and EU2657 but negative for strains EU4855 and EB9505. All strains were isolated from urine cultures except EB9505, which was isolated from blood culture. The isolates were identified by VITEK 2 (bioMérieux, Marcy l’Etoile, France) or Microbact 12E/A (Medvet Diagnostics, Thebarton, Australia).

The MICs by Etest (AB Biodisk, Solna, Sweden) and isoelectric points of β-lactamases in crude extracts are shown in the Table. Polymerase chain reaction (PCR) for CTX-M genes was performed on strains EU17113, EU2673, and EU2657 by using primers CTX-1 and CTX-2 as described by Pai et al. (*4*). This yielded an ≈780-bp product with DNA extracts from strain EU17113 but not the others. The sequence of this product was identical to *bla*_CTX-M-9_ as found in the GenBank database (accession no. AJ416345.1). PCR was repeated for strains EU2673 and EU2657 with a different primer set as described by Gniadkowski et al. (*5*). This produced ≈600-bp products which were identical to *bla*_CTX-M-11_ and *bla*_CTX-M-15_ (accession no. AJ310929.1 and AY463958.1) for EU2673, and *bla*_CTX-M-2_, *bla*_CTX-M-20_ and *bla*_Toho-1_ (accession no. X92507.1, AJ416344.1, and D37830.1) for EU2657.

**Table:** Characteristics of isolates containing CTX-M and plasmid-mediated AmpC β-lactamases

Isolate	Species	MIC (mg/mL)^a^					
		CAZ	FEP	CRO	AZT	pI^b^	Sequenced β-lactamase
EU17113	*K. pneumoniae*	4	4	128	2	7.2, 7.5, 8.0	CTX-M-9 type
EU2673	*K. pneumoniae*	128	128	>256	>256	5.8, 7.2, 7.5, 9.0	CTX-M-15 type
EU2657	*E. coli*	16	128	>256	64	5.1, 6.3, 7.2, 7.5, 8.0	CTX-M-2 type
EU4855	*E. coli*	>256	4	>256	64	8.8	CMY-2 type
EB9505	*E. coli*	128	1	256	8	5.1, 8.8	CMY-2 type

^a^*K. pneumoniae*, *Klebsiella pneumoniae*; *E. coli*, *Escherichia coli*; CAZ, ceftazidime; FEP, cefepime; CRO, ceftriaxone; AZT, aztreonam. ^b^Isoelectric points of β-lactamases.

CTX-M ESBLs can be grouped into four clusters based on the similarity of their amino acid sequences: CTX-M-1 type (CTX-M-1, -3, -10, -11, -12, -15, -23, -28), CTX-M-2 type (CTX-M-2, -4, -5, -6, -7, -20, Toho-1), CTX-M-9 type (CTX-M-9, -13, -14, -16, -17, -18, -19, -21, Toho-2), and CTX-M-8. Three of the four clusters of CTX-M-type enzymes are represented in our small sample of isolates. The only other report of CTX-M enzymes in Southeast Asia of which we are aware describes CTX-M-14 and -17 in Vietnam (*6*).

Diagnostic laboratories may fail to identify CTX-M–positive isolates as ESBL producers if ceftazidime resistance is used as the sole screening criterion, which is unlikely in Singapore because ceftriaxone is usually in the first-line panel for antimicrobial susceptibility testing of *Enterobacteriaceae*. In addition, ESBL screening is conducted in most laboratories by the Jarlier double disk diffusion method (*7*), with at least two different β-lactam disks placed on either side of an amoxicillin-clavulanate disk. However, CTX-M producers may not be distinguished from other ESBL producers because once the ESBL test is positive, the organism is reported as resistant to all third-generation cephalosporins. The importance of the characteristic antimicrobial susceptibility pattern (cefotaxime- or ceftriaxone-resistant, ceftazidime-susceptible) may not be appreciated and may be lost in the edited report. We paid special attention to these isolates because we were evaluating a VITEK 2 at the time and noted the original susceptibility pattern while reviewing the machine reports.

Multiplex PCR for plasmid-mediated AmpC genes was performed on strains EU4855 and EB9505 by using the method described by Perez-Perez and Hanson (*8*). Amplified products of ≈460 bp were produced which, when sequenced, were identical to the gene sequences encoding C-1 molecular subgroup plasmid-mediated AmpC enzymes CMY-2 (accession no. X91840.1), and LAT-3 (accession no. U77414.1) (*9*). This family of enzymes is thought to be derived from the chromosomal β-lactamase of *C. freundii* and includes some of the most widely distributed plasmid-mediated AmpC β-lactamases. CMY-2 has been recently found in *E. coli* strains from Malaysia, which shares a common border with Singapore (*10*).

Although identifying these enzymes has little impact on managing a patient, recognizing CTX-M and plasmid-mediated AmpC enzymes affects antimicrobial drug-resistant surveillance because important new mechanisms of extended-spectrum cephalosporin resistance are represented.

The following sequences have been submitted to GenBank: EU17113 (accession no. AY517474), EU2673 (accession no. AY517476), EU2657 (accession no. AY517475), EU4855 (accession no. AY517473), and EB9505 (accession no. AY514304).
